# Quality and high yield synthesis of Ag nanowires by microwave-assisted hydrothermal method

**DOI:** 10.1186/s11671-015-0774-x

**Published:** 2015-02-06

**Authors:** Manuel F Meléndrez, Carlos Medina, Francisco Solis-Pomar, Paulo Flores, Mani Paulraj, Eduardo Pérez-Tijerina

**Affiliations:** Advanced Nanocomposites Research Group (GINA), Faculty of Engineering, University of Concepcion, 270 Edmundo Larenas, Box 160-C, Concepcion, 4070409 Chile; Hybrid Materials Laboratory (HML), Faculty of Engineering, University of Concepcion, 270 Edmundo Larenas, Box 160-C, Concepcion, 4070409 Chile; Department of Materials Engineering (DIMAT), Faculty of Engineering, University of Concepcion, 270 Edmundo Larenas, Box 160-C, Concepcion, 4070409 Chile; Facultad de Ciencias Físico-Matemáticas, Universidad Autónoma de Nuevo León, San Nicolas de los Garza, Nuevo León 66451 Mexico; Department of Mechanic Engineering (DIM), Faculty of Engineering, University of Concepcion, 219 Edmundo Larenas, Box 160-C, Concepcion, 4070409 Chile; Department of Physics, Faculty of Physical and Mathematical Sciences, University of Concepcion, P.O. Box 160-C, Concepcion, 4070409 Chile

**Keywords:** Nanowires, Hydrothermal synthesis, Nanostructures, Microwave

## Abstract

Silver nanowires (Ag-NWs) were obtained using microwave-assisted hydrothermal method (MAH). The main advantage of the method is its high NWs production which is greater than 90%. It is also easy, fast, and highly reproducible process. One of the drawbacks presented so far in the synthesis of nanostructures by polyol path is the high temperature used in the process, which is superior than the boiling point of solvent (ethylene glycol), and also its excessive reaction time. Here, Ag-NWs with diameters of 70 to 110 nm were synthesized in 5 min in large quantities. Results showed that dimensions and shape of nanowires were very susceptible to changes with reaction parameters. The reactor power and reactor fill capacity were important for the synthesis. It was found that the reaction time needs to be decreased because of the NWs which start to deform and break up due to significant increase in the pressure's system. Energy-dispersive X-ray spectroscopy and electron diffraction analysis (SAED) did not show corresponding phases of AgO. Some aspects about synthesis parameters which are related to the percent yield and size of nanowires are also discussed.

## Background

One-dimensional (1D) silver nanowires (Ag-NWs) are among the most important nanomaterials due to their potential applications in photoluminescence [1], photonic crystals [2], surface-enhanced Raman scattering [3], isotropic conductive adhesives [4], field emission devices [5], high-density magnetic recording devices [6], and sensors [7]. It functions mainly as interconnects or active components in fabricating nanodevices. Control of appropriate shape and size of Ag-NWs can be used efficiently in the diverse applications as mentioned above. Efforts have currently been focused in the control of the synthesis process and assembly of such nanostructures. For this reason, it is important to understand its synthesis process. It has been found that Ag-NWs have exceptional thermal, electrical, and optical properties. Transparent conductive films of Ag-NWs have demonstrated potential to replace indium tin oxide (ITO) in solar cells, displays, touch panels, and organic light-emitting diodes (OLED) [8].

There are many physical [9-15] and chemical [16-20] methods that have been reported for synthesizing Ag-NWs. The percentage yield of the nanowires is an important factor. In most of these processes, nanoparticles and mixtures of other nanostructures are hard to deal with their separation; therefore they limit their use due to different sizes. The use of precursor molecules of preferential growth, like cetyl trimethylammonium bromide (CTAB) or polyvinylpyrrolidone (PVP) sometimes also significantly have proved disadvantageous as they interact strongly with the metal surface, increasing the reaction times and requiring higher temperatures. Moreover, the use of template synthesis to control the diameter and morphology [21] also proves that it is complicated.

Reduction of AgNO_3_ in ethylene glycol, known as polyol method, is the most widely used chemical method to synthesize Ag-NWs, where PVP is used as the precursor molecule of preferential growth. Xia et al. [22] and Sun et al. [23] improved this method and obtained Ag-NWs with high aspect ratios at a well-defined reactant addition rate in a dried ethylene glycol system. Low precursor concentrations and a slow addition in the reaction medium forms multiply twinned particles (MTPs) just when the nucleation process starts, and these particles serve as seeds to grow silver nanowires [24].

High-purity, uniform, and smaller Ag-NWs can also be obtained from the modified polyol method which also increases the yield percentage. Adding redox active metal salts like (Fe^II/^Fe^III^ or Cu^I^/Cu^II^) [25,26], Na_2_S [27], and NaCl [28] or introducing Pt seeds in the presence of PVP [29] facilitates a controlled growth of nanowires. This occurs because of the NWs which are grown from multiple-twinned seeds with a decahedral structure, while seeds with a single-crystal structure tend to evolve into polyhedral nanocrystals with a cubic or octahedral shape. Therefore, for producing high yield, it becomes a necessity to develop an efficient way to promote the formation of multiple-twinned seeds at the nucleation stage during the synthesis [30]. Investigations on synthesis mechanism elucidates that these variable valency metal ions having low valence can remove oxygen from the solvent preventing twinned seeds that are dissolved by oxidative etching during the initial formation of seeds, thereby scavenging absorbed atomic oxygen from the surface of seeds. For this reason, the formation of AgCl colloid results in slow releasing of Ag^+^ in the solution, which facilitates the formation of Ag-NWs [31]. However, it is important to mention other works that have been focused on improving the size, controlling the shape, increasing the yield percentage, and reproducibility of the reaction to obtain high-purity silver nanowires that are still ongoing as there has not been found an efficient method.

In this work, we report a microwave-assisted hydrothermal method where microwaves directly heats the volume of liquid while the environment or the containers are heated only by reaction's mixture, in order to achieve high-quality and yield Ag-NWs. As molecules with dipolar structure (e.g., ethylene glycol, PVP) oscillate in a microwave fluctuating field, this oscillation generates a molecular movement resulting in friction leading to heat. Another advantage is that molecules having an ionic structure (e.g., AgNO_3_) get aligned in the electromagnetic field facilitating a favorable nucleation and growth processes of the 1D nanostructures. This method significantly reduces reaction time compared to the conventional heating time, whereby yielding high-quality, narrow size good-shaped Ag-NWs, and reproducibility is very high in this method.

## Methods

### Synthesis

Ag-NWs were synthesized by microwave-assisted hydrothermal method (MAH). In this process, silver nitrate (AgNO_3_), PVP, and ethylene glycol purchased from Sigma Chemicals (St. Louis, MO, USA) were used without further purification. Separate solutions of 0.1 mol/L (AgNO_3_) and 0.6 mol/L (PVP) were prepared in anhydrous ethylene glycol. Each solution was stirred to complete the dissolution in a dry box under nitrogen atmosphere. Then, they were transferred into a Teflon vessel of 23 mL capacity microwave transparent body autoclave. The reactor was kept closed inside the dry box that was placed inside a conventional oven under a regulated power of 200 to 1,000 W. In order to prevent overpressure inside the reactor, the synthesis process was carried out by power pulses of 1.0 min and relaxation pulses of 0.5 min. The first relaxation pulse was for 0.5 min followed by a 1.0-min pulse. The reaction time to the total time of pulses with power for each treatment was between 5.0 and10 min. At the end of the reaction, the reactor was cooled down to room temperature. Cotton-like appearance was formed at the top of the reactor chamber; this was carefully removed with a spatula and was washed with deionized water and ethanol several times to remove the excess PVP from the Ag-NWs surface. The resulting material was rich in Ag-NWs of size approximately 70 to 110 nm. The residue left at the bottom of the reactor (approximately 5%) was discarded. Reactor power, reactor fill capacity, and molar ratio (PVP: AgNO_3_) were the parameters carefully considered in the synthesis.

### Characterization

Scanning transmission electron microscopy (STEM) was performed in a probe-corrected JEOL-JEM-ARM 200 F (JEOL Ltd., Akishima-shi, Japan) operated at 200 kV (point resolution of 0.08 nm). Scanning electron microscopy (SEM) was performed using a FEG Hitachi S-5500 ultra high-resolution scanning electron microscope (0.4 nm at 30 kV; Hitachi Ltd., Chiyoda-ku, Japan) with a BF/DF Duo-STEM detector.

## Results and discussion

**Figure 1:**
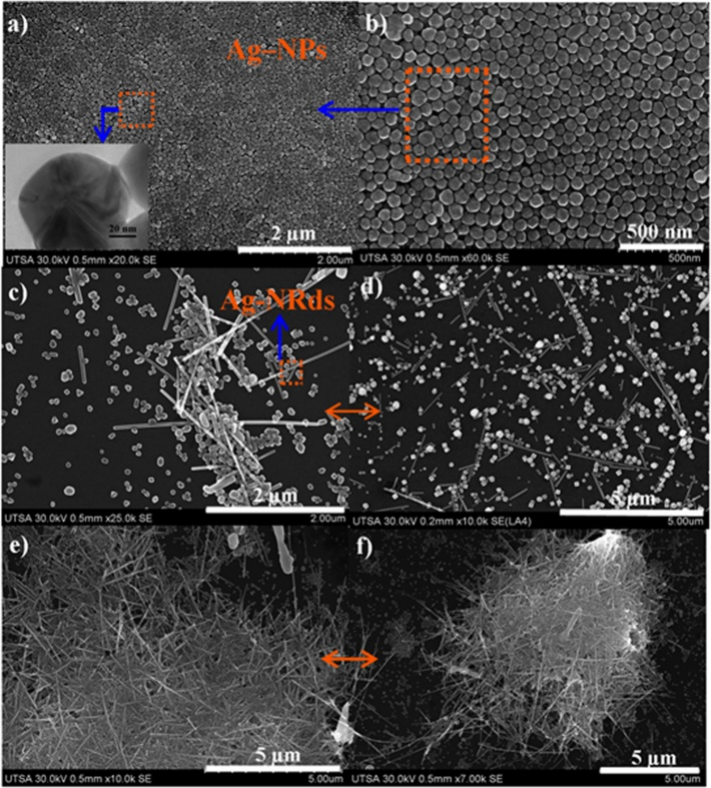
**Micrographs of Ag-NWs and NPs obtained through MAH for 20% reactor filling capacity.** Treatments with different powers: **(a, b)** 200 W; **(c, d)** 400 W; **(e, f)** 600 W.

**Figure 2:**
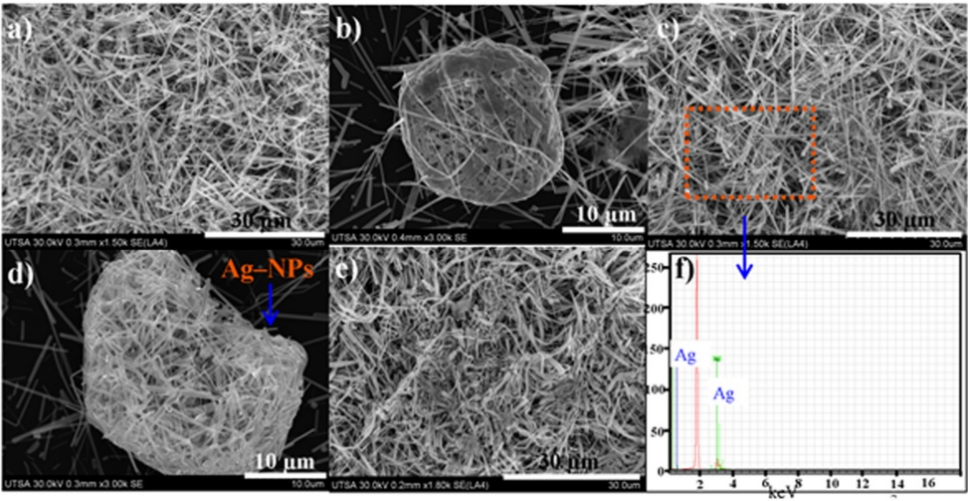
**Ag-NWs images obtained for 40% of reactor filling capacity at 1,200 W and for different reaction times. (a, d)** 5 min. **(b, e)** 10 min. **(c, f)** EDX of Ag nanowires obtained at 10 min.

**Figure 3:**
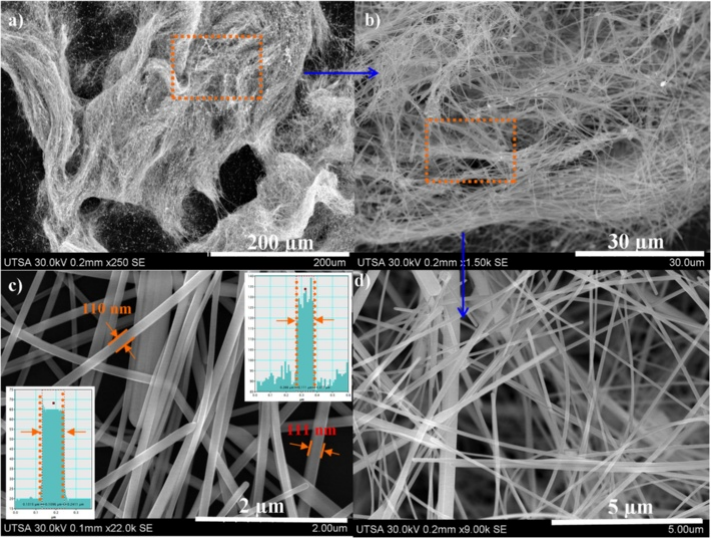
**Ag-NWs images obtained for 40% of reactor filling capacity at 800 W. (a, b)** Ag nanowires before washing process. **(c, d)** After washing process.

**Figure 4:**
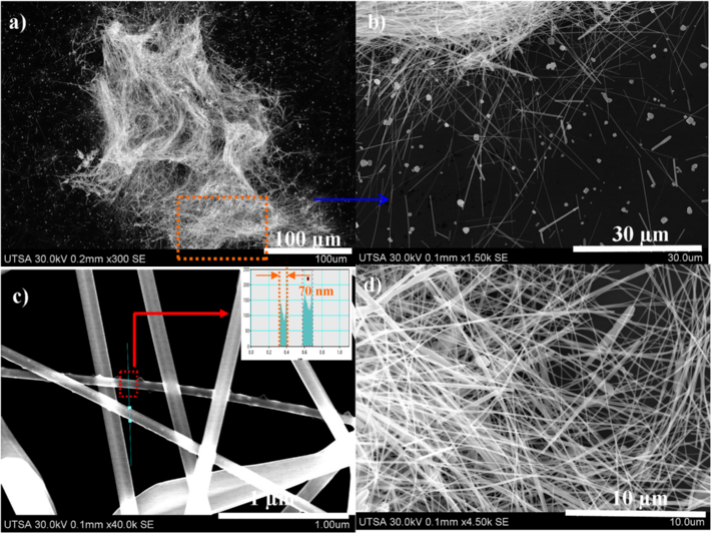
**Ag-NWs images obtained with a 20% of reactor filling capacity at 800 W. (a, b)** Ag nanowires before washing process. **(c, d)** After washing process.

**Figure 5:**
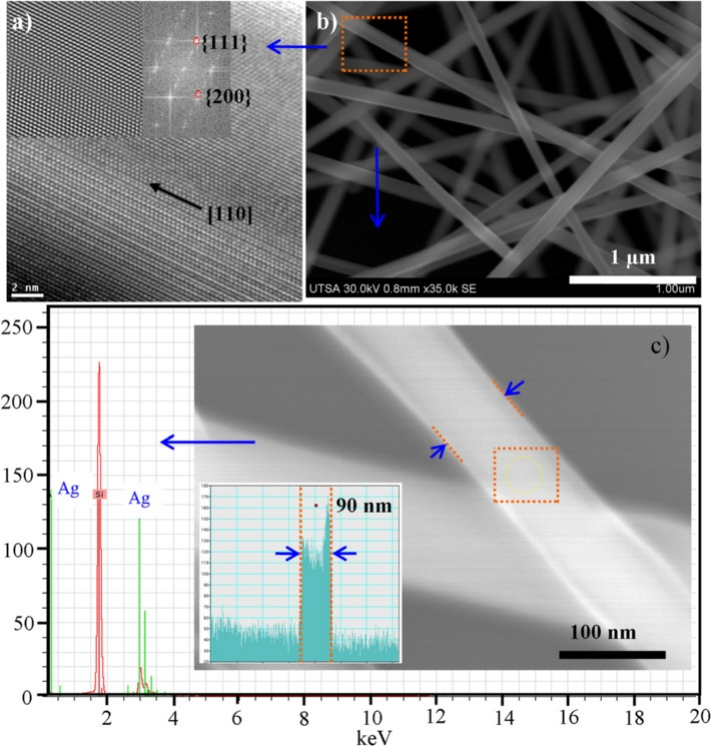
**FFT of plane area in the HRTEM image. (a)** Fast Fourier transformation (FFTs) image of Ag-nanowires lattice fringes. **(b)** Ag-nanowires synthesized at 800 W, 40% of reactor filling capacity, 5 min. of reaction time. **(c)** EDX of Ag-NWs.

MAH was a quick method compared to the conventional hydrothermal method, since the conventional methods require a reaction time exceeding 2 h and temperatures above 160°C. MAH is a fast method and economical due to the reaction time of the process which is not greater than 10 min. The main disadvantage in the conventional hydrothermal method was to take care of a number of synthesis parameters in order to improve the yield, which was avoided in this method. Ag-NWs synthesized using MAH method are shown in Figures 1, 2, 3, 4, and 5; all reactions were performed by using the following molar relation (1:6) AgNO_3_ (0.1 mol/L) and PVP (0.6 mol/L), respectively. The above molar relation was established in preliminary tests with different reactant ratios and concentration by using various microwave powers. The 1:6 reagent relation was the most efficient to obtain Ag-NWs of high quality. PVP must always be in excess because it acts as a precursor molecule for preferential growth of NWs. If the molar ratios (1:6) were maintained and the reactant concentration increases during a typical synthesis, the reaction product rich in Ag nanoparticles will be obtained due to the strong interaction of PVP with the silver seed surface, avoiding the preferential growth to obtain 1D nanostructures. This situation is favored by the large amount of PVP molecules that are present in the solution and also preventing the diffusion of Ag^2+^ species to the active growth centers. Figure 1 shows micrographs of Ag-NWs obtained for 5 min of reaction time and 20% of reactor fill capacity, respectively. Different reactor powers 200 W (Figure 1a,b), 400 W (Figure 1c,d), and 600 W (Figure 1e,f) were used in these experiments. When the reaction was carried out using small reactor power, homogeneous silver nanoparticles were obtained with sizes ranging between 50 and 70 nm. Instead, if the reactor power is raised to 400 W, then the mixture of the nanoparticles and nanowires of lesser proportion is produced. Particle size in this treatment ranged between 60 and 90 nm. Ag-NPs were slightly larger compared to those obtained with 200 W. NWs had lengths above 5 μm with diameters about 90 nm. When the reaction was performed at 600 W, the NWs proportion compared to the NPs was found to be greater. These had the same dimensions than those synthesized at 400 W. In conventional experiments, it was not possible to obtain high production of Ag-NWs. NPs and NWs mixture were always obtained except for treatment at 200 W, which only produces NPs. This may be due to inadequate pressure inside the chamber that would not have been enough for Ag^2+^ ion migration towards the active growth seeds, which also interacts with PVP that induces preferential growth for nanostructures. Unlike conventional heating, microwave directly heats the volume of the liquid while leaving the surroundings (i.e., the containers) untouched. Moreover, the dipolar molecules, e.g., ethylene glycol and PVP oscillates due to microwave fluctuating field. This oscillation generates a molecular movement resulting in friction and therefore generates heat. The reactor was placed in a fixed position in the microwave oven; for this reason, molecules having an ionic structure (e.g., AgNO_3_) get aligned along the electromagnetic field, according to the above, with the rapid heating of the solution, nucleation and growth process for 1D nanostructures is facilitated. MAH provides a combination of rapid and efficient heating which overheats the solvent above its boiling point, and this leads to obtain products much faster. To achieve overheating, a considerable reactor power is needed to increase the molecular friction above the boiling point of solvent. Therefore, temperature and pressure of the synthesis process is not just enough to induce nanowire growth. Figure 1a,b,c,d,e,f shows that on increasing reactor power, the Ag-NWs production improves substantially. With these reaction conditions, reaction time was not sufficient for full conversion of silver seed into nanowires.

Experiments using both higher reactor power (1,200 W) and reactor fill capacity (40%) were also performed and is also shown in Figure 2a,b,c,d,e,f. Figure 2a,d and b,e corresponds to experiments carried out with different reaction times at 5 and 10 min, respectively. In the micrographs, it can be observed that a considerable increase, the reactor power leads to Ag-NWs of more than 30 μm in length and diameters ranging between 90 and 150 nm. Most of the NWs were found to intertwine forming agglomerates, as seen in Figure 2b,d. These structures were different from those obtained for 600 W. Ag-NWs synthesized with this reactor power were not found to intertwine or agglomerate but had smaller diameter. An increase in the pressure due to overheating of solvent was produced for 1,200 W. This reactor power produced Ag-NWs well before 5 min. Therefore, by raising reaction time, the Ag-NWs continue to grow until the ions in the solution gets depleted. Owing to high pressure in the reaction chamber, they begin to degrade leading to intertwines and agglomeration, as seen in Figure 2b,d. Increasing reactor power is counterproductive to obtain well-shaped and homogeneous silver nanowires. Characteristic picks of Ag metal appearing in the spectrum at 2,984 KeV [Ag(*L*α)] and 3.151 KeV [Ag (*L*β)] observed in EDX analysis (Figure 2f) reveals that the NWs composition consists only of silver atoms.

To prevent degradation and intertwining of Ag-NWs as in previous cases, synthesis was performed at 800 W using two reactors fill capacity of 40% and 20%. Results obtained are shown in Figures 3 and 4, respectively. High production of Ag-NWs at 40% reactor fill capacity was observed because a possible explanation is that Ag^2+^ ions get reduced during the synthesis. NWs are then separated by decantation of the reaction solution. Ag-NPs get dispersed in the supernatant solution. After washing and drying under reduced pressure, a greater yield of 85% was obtained (Figure 3c,d). These NWs were above 50 μm and diameters ranging between 70 to 110 nm. They were not found to be intertwined as those obtained at 1,200 W (Figure 2b,d). Reaction time (5 min) was found to be ideal for appropriate preferential growth; in these experiments, it was observed that an excessive reaction time and reactor power are counterproductive in Ag-NWs synthesis. In order to optimize solvent suitable to produce an appropriate saturation pressure to achieve better performance and homogeneity of the NWs, it is important to try different reaction times and reactor powers. The reactor fill capacity in combination with the reactor power also affects the NWs dimensions, as these parameters are directly related to the system pressure. Figure 4 shows Ag-NWs obtained for 800 W and 20% of reactor fill capacity. These NWs were found to have smaller diameters than those obtained for 40% of reactor fill capacity, shown in Figure 3. Production declines on obtaining higher proportion of nanowires and nanoparticles mixture. In the MAH as well as in the conventional heating method, the following is evident: if one wishes to increase the yield percent of nanowires, the synthesis parameters must be increased. This leads to obtain nanomaterials larger in diameter and length. Conversely, a reduction in the parameters leads to lower dimensions and also low production. This is mainly owing to the system pressure which is very sensitive to the changes in the reactor power, reactor fill capacity, precursor's concentrations, and products formed in the reaction.

**Figure 6:**
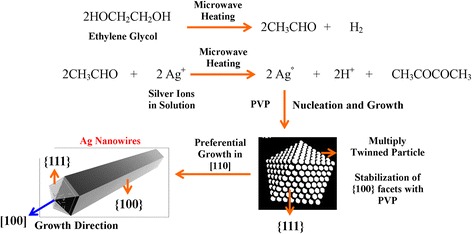
**Proposed mechanism for the synthesis of silver nanowires through microwave-assisted hydrothermal method (MAH).**

HRTEM analysis of nanowires obtained at 800 W and 5 min was performed in order to determine its structure. These materials tend to grow as penta twinned at {111} planes. Figure 5a shows a FFT of plane area in the HRTEM image. The pattern clearly shows that there is a superposition of more than one diffraction pattern. The FFT corresponds to the diffraction pattern of penta-twinned NWs [32], corresponding to a superposition of [100] and [112] zone axes (double check). The calculated spacing distance was 2.35 and 2.03 Å, which correspond to (111) and (200) planes of metallic silver (JCPDS file No. 04–0783 from ASTM), respectively. Analysis revealed that Ag-NWs have a preferential growth in the [110] direction (along the fivefold axis), shown in Figure 5a. This is confirmed also because EDX analysis revealed that these were only constituted by silver atoms (2.984 keV [Ag (*L*α)] and 3.151 keV [Ag (*L*β)]). Phases corresponding to AgO were not found; thereby, possible oxidation of the nanowires was discarded. The growth mechanism of the current MAH method is deduced to be similar to that previously described for the polyol process. It is important to consider that the reaction times with the MAH method are smaller, which prevents that the increment of the hydronium ions concentration degrades the silver seeds and thereby prevent that percentage of nanowires obtained in the process is reduced. In Figure 6, a diagram of possible growth mechanism silver nanowires by the MAH method is shown [33]. The mechanism steps are: *i*) the reduction of silver ions, *ii*) the formation of multi-twinned-crystal seeds, and *iii*) the growth of seeds into nanowires. In the first step, Ag^+^ ions are reduced to Ag° atoms by ethylene glycol [34]. In the second step, the adsorption of PVP on the Ag passivates the more active {100} plane and also acts as the driving force to form cyclic penta-twinned crystal seeds [23]; the penta-twinned nanocrystals play a key role in the confinement of the diameters of the nanowires and the growth of the longitudinal direction. In the last step, due to much stronger chemical bonding between PVP and {100} compared to that between PVP and {111}, the {111} facet remains active, resulting in longer wire with a pentagonal cross section. As mentioned above, the advantage of the MAH method is based on reducing the growth times to prevent the degradation of the penta-twinned-crystal seeds due to the acid formed in solution. In this method, the system pressure increases rapidly; this variable is closely related to the reactor power, reactor fill capacity, reaction time, and molar ratio. Therefore, the growth is very fast so that the nanowires diameter could be higher compared to other open system wet methods.

One of the major problems that arise in the application development of nanomaterials is controlling the size and shape of the nanostructures which, due to any temperature gradient, may lead to obtain a mixture of shapes and sizes of these. Another problem that arises is the amount of synthetic material. For that reason, with MAH method, it is possible to obtain silver nanowires fast and with high yield percentage. These materials can be used in the manufacture of optoelectronic devices; also, due to its surface plasmon resonance effect (SPR) [35], these can be used in the molecule detection and the manufacture of specific biosensors [36], isotropic conductive adhesives, solar cells, displays, touch panels, and organic light-emitting diodes, on-chip interconnects, and other typical applications of 1D nanostructured materials [37,38].

## Conclusions

High yield of Ag-NWs were obtained by the MAH method. NWs diameter synthesized through this method ranged from 70 to 110 nm in diameter. Percentage rate of NWs depended largely on the synthesis parameters, especially, reactor power and reactor fill capacity. Higher yield of Ag-NWs was achieved at 800 W, 5 min of reaction time, and with 40% of reactor fill capacity. It was found that an excessive reactor power considerably deformed nanowires and produces large diameter and a lot of agglomerates of NWs, making the separation and purification process harder. In comparison to conventional hydrothermal method to produce Ag-NWs, MAH method produces nanowires of diameter greater than 50 nm; moreover, its productivity rate is also high.
